# Optimization of Mechanical Properties for Polyoxymethylene/Glass Fiber/Polytetrafluoroethylene Composites Using Response Surface Methodology

**DOI:** 10.3390/polym10030338

**Published:** 2018-03-20

**Authors:** Jasbir Singh Kunnan Singh, Yern Chee Ching, Luqman Chuah Abdullah, Kuan Yong Ching, Shaifulazuar Razali, Seng Neon Gan

**Affiliations:** 1 Department of Chemical Engineering, Faculty of Engineering, University Malaya, Kuala Lumpur 50603, Malaysia; jasbi@siswa.um.edu.my; 2 Department of Mechanical Engineering, Faculty of Engineering, University Malaya, Kuala Lumpur 50603, Malaysia; shaiful@um.edu.my; 3 Department of Chemical Engineering, Faculty of Engineering, University Putra Malaysia, Serdang 43400, Malaysia; chuah@upm.edu.my; 4 School of Foundation, University of Reading Malaysia, Persiaran Graduan, Kota Ilmu, Educity, Iskandar Puteri Johor 79200, Malaysia; kuanyong84@hotmail.com; 5 Department of Chemistry, Faculty of Science, University Malaya, Kuala Lumpur 50603, Malaysia; gansn@yahoo.com

**Keywords:** polyoxymethylene, polytetrafluoroethylene, surface etch, RSM, optimization

## Abstract

This paper investigated the effects of polytetrafluoroethylene (PTFE) micro-particles on mechanical properties of polyoxymethylene (POM) composites. Since PTFE is immiscible with most polymers, the surface was etched using sodium naphthalene salt in tetrahydrofuran to increase its surface energy. The effects of two variables, namely PTFE content and PTFE etch time, on the mechanical properties of the composite were studied. Experiments were designed in accordance to response surface methodology (RSM) using central composite design (CCD). Samples were prepared with different compositions of PTFE (1.7, 4.0, 9.5, 15.0, or 17.3 wt %) at different PTFE etch times (2.9, 5.0, 10.0, 15.0, or 17.1 min). Four mechanical properties of the POM/GF/PTFE composites, that is, strength, stiffness, toughness, and hardness, were characterized as a function of two studied variables. The dependency of these mechanical properties on the PTFE etch conditions was analyzed using analysis of variance (ANOVA). Overall desirability, D global index, was computed based on the combination of these mechanical properties for POM/GF/PTFE composites. The D global index was found to be 87.5%, when PTFE content and PTFE etch time were 6.5% and 10 min, respectively. Good correlation between experimental and RSM models was obtained using normal probability plots.

## 1. Introduction

Polyoxymethylene (POM) is an excellent engineering thermoplastic well known for its superior tribological properties and good balance of mechanical and thermal characteristics [1,2]. Attempt to improve one of these properties usually results in deterioration of another. Improvements in mechanical and tribological properties are typically carried out by blending with other polymers [3,4], fibers [5,6], and micro- or nano-sized particles. The fibers and particles used as modifiers can be organic or non-organic [7,8,9].

On the contrary, POM has very poor compatibility with other materials. Compatibilizers are often used as additives to obtain the desired properties of its composites [10]. Improving the compatibility of immiscible polymers results in improved morphology and properties of the composite [11,12,13]. It is often challenging to disperse fillers effectively in the matrix polymer of a composite. Development of compatibilization technologies will be crucial for the polymer industry to reap the full benefits of such approaches to obtain materials with optimum performance and cost characteristics.

The addition of glass fibers (GF) to POM as reinforcement has been one approach to improve strength, stiffness, and hardness. The change of these properties is due to the strength of GF holding the POM matrix together and the bond of GF to the POM matrix. When impacted or loaded, the energy absorbed by the reinforcement makes the polymer not only tougher, but also stronger. This is evident when comparing the morphology of a fractured surface for filled versus reinforced after impact testing. Addition of GF to POM negatively affects the wear resistance and coefficient of friction (COF) [14,15].

POM have good self-lubricating characteristics with low coefficient of friction (COF) coupled with high wear resistance. However, pure POM may not be able to meet the requirement of an application by depending on its own inherent properties only. As such, the addition of particulates is necessary in applications where tribological and mechanical properties are of equal importance, Typically, tribology properties are enhanced by blending POM with solid lubricants such as molybdenum disulfide (MoS_2_), alumina (Al_2_O_3_) and polytetrafluoroethylene (PTFE) in micro- or nano-sized particles and other polymeric materials such as polyethylene oxide (PEO), polylactic acid (PLA), etc. [7,8,9,10,12,13,16,17].

PTFE is a hydrophobic polymer often used for wear and COF reduction in thermoplastics. Its composites are typically processed using melt mixing process. Since the surface energy of PTFE is low, melt mixing process leads to poor distribution and non-homogenous dispersion. In composites where PTFE is added as second or third phase, mechanical properties can be compromised depending on the matrix material [18,19]. As such, surface modification of PTFE is required to enhance compatibility to the matrix. The methods commonly employed to alter the chemical structure of PTFE are chemical etching, electron beam irradiation, and plasma treatment [20,21].

Naturally, adhesion between POM and PTFE is poor due to incompatibility between these two polymers. PTFE has very low wettability and bond ability due to its low surface energy and non-stick properties [11]. Its chemical stability and inertness makes the surface modification of PTFE very challenging. In order to impart polar functional groups to form hydrogen, oxygen, or other bonds with the backbone carbon chain, the surface needs to be etched chemically. One of the conventional techniques applied is treatment using an alkaline metal solution that de-fluorinates the surface [22].

Suresha et al. [16] reported that the addition of PTFE particles into neat POM deteriorated the tensile strength by 23%. Addition of glass fibers improved the strength of POM/GF/PTFE composite by 20%. Tribology characterization was not reported. Benabdallah reported that POM filled with 20% GF resulted in deterioration of COF and wear resistance. When POM was filled with 20% PTFE micro powder, COF and wear resistance improved significantly [14]. Franklin et al. [23] conducted a detailed study investigating the relationship between the characteristics of a transfer layer formed by POM filled with 20% PTFE and the counter face surface topography. Wear rate was influenced by the counter face surface topography and the characteristics of the transfer layer. Mechanical properties such as tensile strength, elasticity modulus, elongation at break, and impact strength can be improved as a result of better compatibility of POM and PTFE. This is evident in the work carried out by Chiang et al. [11], where increasing PTFE particles composition up to 15% steadily improved tensile properties. Beyond 15% composition, the strength of POM/PTFE composite deteriorated. With surface modification of PTFE particles through chemical etching, superior mechanical properties were achieved. Huang et al. [24] characterized the effects of interface modification between POM and PTFE in the form of fibers. The surface of PTFE fibers was treated first using argon plasma and then grafted with acrylic acid. The impact strength, coefficient of friction, and wear resistance of the composite by blending POM with surface modified and grafted PTFE fibers were double that of non-treated PTFE fibers.

It is known that toughening of polymer composites by addition of fillers in the form of particulates usually results in reduction of strength and stiffness. Addition of fibers can compensate for the loss of these mechanical properties. Where tribological properties are of equal importance, a suitable particulate filler can be added to the composite. To obtain a high-performance POM composite, the dependency of mechanical properties against the variation of fillers during sample fabrication is vital.

In this work, POM/GF is used as a matrix where GF acts as the reinforcement phase. The composition of GF is unchanged at 25% of weight ratio. Surface modified PTFE microparticles are melt-blended with the matrix. The POM/GF/PTFE composites’ strength, stiffness, toughness, and hardness are characterized with PTFE content and PTFE etch time as control variables. Response surface methodology (RSM) is employed to determine the dependency of these mechanical properties against PTFE content and PTFE etch time. The aim of this work is to identify a stable region where the mechanical properties for POM/GF/PTFE composites are optimal. Based on the literature search, there is no information available on any optimization work involving POM/GF/PTFE composites using the statistical modeling approach.

## 2. Materials and Methods

### 2.1. Materials

Polyoxymethylene (POM) with 25% GF reinforcement matrix material used in this work purchased from Du Pont (Starke, FL, USA), commercially known as POM525GR. It is a homopolymer with a density of 1.6 g/cm^3^ and a melting temperature of 178 °C. Polytetrafluoroethylene (PTFE) microparticles with an average particle size of 12 µm, density of 0.425 g/cm^3^, and specific surface area of 1.5–3.0 m^2^/g were also purchased from Du Pont. The PTFE etch solution was prepared in the lab using sodium naphthalene with a density of 0.45 g/cm^3^. Tetrahydrofuran, purchased from J.T. Bakker, (Phillipsburg, NJ, USA) was used as a solvent to dissolve the salt.

### 2.2. Preparation of PTFE Microparticles Etch Solution

The etch solution was prepared by stirring sodium naphthalene in tetrahydrofuran at 25 °C at a stirring speed of 350 rpm. The mixture was comprised of 5% of sodium naphthalene to 95% of tetrahydrofuran and was stirred for 5 min, resulting in a dark brown solution. Subsequently, 30 g of PTFE microparticles were added to the solution and stirred at 25 °C with a stirring speed of 525 rpm. The stirring time was varied to obtain different surface etch depths. Once the required etching time was achieved, the stirrer was shut off and the solution was left to settle through sedimentation for about 1 min. The solid sediment containing PTFE and sodium salt settled at the bottom, separated from the liquid. The upper liquid was then poured away carefully.

For each wash cycle, 200 cm^3^ of acetone was added to the PTFE and stirred for 5 min with 525 rpm. Upon completion of the first wash cycle, the solution was left to settle, followed by pouring away the upper liquid. Another two wash cycles were repeated. The process was then followed by rinsing of the solid using 200 cm^3^ of distilled water. Stirrer speed of 525 rpm was used for rinsing. After each rinse cycle, the solid was left to settle and the upper portion containing mainly dissolved sodium salt in distilled water was poured away prior to the next rinse cycle. A total of five rinse cycles were performed to completely separate the etched PTFE micro powder from the sodium salt. The residue containing only the etched PTFE micro powder in distilled water was then placed in a petri dish of 150 mm diameter. This formed a PTFE layer of approximately 1 mm thick. An incubator with a temperature of 40 °C was used to dry the solution for 48 h. The etched PTFE was then removed from the petri dish and placed in a lab container in a dark environment to prevent exposure to light.

### 2.3. Preparation of POM Composites

POM525GR and surface-etched PTFE with various mix ratios by weight were compounded by melt blending using a Brabender Mixer 50EHT 3Z (Brabender GmBH & Co KG, Kulturstraße, Duisburg, Germany). Processing parameters of the mixer were temperature 180 °C, blades rotational speed 60 rpm and 10 min mix time. It was then crushed to approximately 1–3 mm in length prior to an injection molding process using a BOY XS machine (BOY Machines, Inc., Exton, PA, USA). The molding was comprised of three main processes, i.e., filling, plasticizing, and holding. For the filling process, the injection pressure used was 14 MPa with an injection speed of 100 mm/s. For the plasticizing process, pressure, screw rotational speed, and barrel temperature were controlled to 1 MPa, 170 rpm, and 180 °C, respectively. A holding pressure of 12 MPa was applied during the melt injection into the mold.

### 2.4. Model Development Using Response Surface Methodology (RSM)

RSM is a combination of mathematical and statistical techniques. These techniques are useful for the modeling and analysis of problems where the response is influenced by several input variables [25,26,27]. RSM is able to quantify the relationships of the response (Y) to the input variables ($x_{1},\;x_{2},\;\ldots ,\;x_{k}$). If these input variables are determinable, randomized on the experiment, and with minimal error, the response (Y) can be expressed as:

(1) $$Y=f\left(x_{1},\;x_{2},\;\ldots ,\;x_{k}\right)+\varepsilon$$

The input variables are transformed into coded values and are determined using the following equation:

(2) $$x_{i}=(X_{i}-X_{0})/∆x$$

where $x_{i}$ is the coded value for the *i*-th variable, $X_{i}$ is the uncoded value of the *i*-th variable, and $X_{0}$ is the uncoded value of the *i*-th variable at the center point. The regression analysis is performed to estimate the response function as a second-order polynomial;

(3) $$Y=\beta_{0}+\overset{k}{\underset{i=1}{\sum }}\beta_{i}x_{i}+\overset{k}{\underset{i=0}{\sum }}\beta_{ii}x_{i}{}^{2}+\overset{k-1}{\underset{i=1,}{\sum }}0\overset{k}{\underset{i<j,\;j=2}{\sum }}\beta_{ij}x_{i}x_{j}+\varepsilon$$

where Y is the predicted response, $\beta_{0}$ is constant, and $\beta_{i}$, $\beta_{ii}$, and $\beta_{ij}$ are the linear, quadratic, and interactions coefficients estimated from the regression design, respectively.

The central composite design (CCD) was used to study the effects of PTFE content and PTFE etch time on the mechanical properties of POM/GF/PTFE composites. Subsequently, the effects of these key process input variables (KPIVs) on their responses or key process output variables (KPOVs) were used in optimization studies. Samples were tested for tensile strength, elasticity modulus, toughness, and hardness. This method suitably fitted a polynomial and optimized the effective input variables to obtain the desired responses. The correlations between these parameters were analyzed as well.

The data collected were analyzed in a statistical manner to determine the relationship between the input variables and each of the responses. To model the response as a mathematical function where the independent input variables may include linear, interaction, quadratic, and cubic terms, a regression design was used. The goal was to obtain good model parameter estimates.

The CCD was built up from two level full factorial design with center points and axial points with one additional center point. These axial points or augmented points were chosen as ±√2 since two factors were of interest. Thus, the experiment comprised 13 runs consisting of four axial points, four high and low levels of factors, and five central points. The values of each input variables were defined at the low, mid, and high points. Table 1 shows the selected KPIVs and their range.

### 2.5. Statistical Analysis and Model Fitting

Statistical analysis, comprising regression and graphical analysis, was performed using Design-Expert software (version 10.0.6, Stat-Ease, Inc., Minneapolis, MN, USA). Based on the regression equation, optimum values of input variables were obtained. Analysis of variance (ANOVA) was used to further justify the adequacy of the models. The procedure calculates F-ratio, the ratio between the regression mean square and the mean square error. The F-ratio, called the variance ratio, is the variance ratio due to the effect of a factor and variance due to the error term. This ratio measures the significance of the model with respect to the variance of all terms included in the error term. The desire is to obtain a model that is significant.

Testing for significance of individual model coefficients forms the basis for optimizing the model. This is achieved by adding or deleting coefficients through forward addition, backward elimination or stepwise elimination, addition, or exchange. *p*-value or probability of risk to falsely rejecting a given hypothesis is determined. Generally, a lowest-order polynomial is chosen to adequately describe the system.

Lack-of-fit is a special diagnostic test for the adequacy of a model. As replicate measurements are available, a test indicating the significance of the replicate error in comparison to the model dependent error can be performed. This test splits the residual or error sum of squares into two portions, one due to pure error based on the replicate measurements and the other due to lack-of-fit based on the model performance. The test statistic for lack-of-fit is the ratio between the lack-of-fit mean square and the pure error mean square. As stated previously, this F-test statistic can be used to determine whether the lack-of-fit error is significant. Insignificant lack-of-fit is desired as significant lack-of-fit indicates that there might be contributions in the input variables–response relationship that are not accounted for by the model.

In addition, verification is needed to determine whether the model actually describes the experimental data. The two basic components of a valid regression model are the deterministic portion and stochastic error. The deterministic portion is the predictor variables in the model. The expected value of response is a function of these predictor variables. Stochastic error is the difference between the actual and predicted values represented by residuals. The residuals must be unpredictable and centered on zero throughout the range of predicted values. Random errors produce residuals that are normally distributed. Therefore, the residuals are in symmetrical pattern and have a constant spread throughout the range. A normal probability plot of residuals tests the dataset in the model to see if it fits a normal distribution. Once residual analysis validates no biased results, statistical measures for goodness of fit between experimental and predicted is performed. The coefficient of determination, *R*^2^, signifies the level of fit of the polynomial model, with values between 0 and 1. *R*^2^ is one of the measures for variability reduction of a response in statistical modeling. As more terms are added, the value of *R*^2^ increases without consideration of the statistical significance of these additional terms. The goal is to obtain *R*^2^ values close to 1. Adjusted *R*^2^ (*R*^2^_adj_) takes into consideration only the terms that are statistically significant. A lower value of *R*^2^_adj_ than *R*^2^ indicates no necessity of adding extra terms into the model.

Adequate precision is a measure of the signal to noise ratio. A precision of more than 4.0 is desired, proving the model is able to predict the response. Then, the model can be used to navigate the design space. The adequacy of the model is investigated by the examination of residuals. The residuals, which are the difference between the observed responses and the predicted responses, are examined using normal probability plots. For an adequate model, the points on the normal probability plots form a straight line. For a weak model, residuals versus the predicted response plots have no obvious patterns.

### 2.6. Optimization of Mechanical Properties Using the Desirability Method

Desirability method was used to determine the values of input variables, i.e., PTFE content and PTFE etch time for optimization of multiple responses, i.e., mechanical properties of POM/GF/PTFE composites simultaneously. The condition of each mechanical property (Y) is selected based on its importance by selecting a maximum, minimum, or a target value of specification. Equation (4) is used to obtain the *D* global index for the overall desirability based on the combination of responses processed through a geometric mean:

(4) $$D={\left(d_{1}\left(Y_{1}\right)\times d_{2}\left(Y_{2}\right)\times d_{3}\left(Y_{3}\right)\times \ldots \times d_{n}(Y_{n})\right)}^{1/n}$$

The responses ($Y_{1}$, $Y_{2}$, $Y_{3}$, $\ldots ,\;Y_{n}$) are transformed such that $0<d_{i}<1$. The d value increases when the *i*-th response approaches the desired condition. Resulting from the geometric mean, *D* evaluates the levels of the combined responses with an index of 0<D<1. It is maximized when all responses approach the desirable specification. Responses can be assigned different importance. All responses with their own importance are included in one desirability index. Multiplication causes an outcome to be low if any one response is unable to achieve its desirability.

The Design-Expert software allows the input variables and responses to be changed to obtain the greatest overall desirability. These input variables are left within their experimental range and only responses are adjusted. This is where subject matter expertise and engineering knowledge become essential. The software also has an option to assign a weighting on a 1 to 10 scale and importance using a five-point scale. In this work, the same weighting was assigned to all mechanical properties. The stiffness and hardness of the POM/GF/PTFE composites were of higher importance than the strength and toughness.

### 2.7. Morphology Analysis Using Scanning Electron Microscopy (SEM)

SEM images provide information on the effects of etching to the surface of PTFE. The morphology of POM/GF/PTFE composites as a result of brittle fracture during tensile testing was also investigated. The central section of the dumbbells was selected for morphology analysis. The micrographs were taken using a Phenom ProX desktop SEM (Phenom-World B.V., Eindhoven, The Netherlands) operated at 10 kV accelerating voltage.

### 2.8. Mechanical Testing

Injection molded samples were tested for tensile strength, elasticity modulus, and toughness using an Instron 3369 universal tensile test machine (Instron, Norwood, MA, USA) according to ASTM D638. Type IV dumbbell-shaped specimens was prepared for tensile tests. Prior to testing, samples were conditioned in accordance with ASTM D618. The crosshead speed is fixed at 5 mm/min at room temperature with the distance between grips of 60 mm. The thickness and width of each sample were measured individually to obtain an accurate cross-sectional area. The average value of six samples for each POM composite type was used to determine its strength, stiffness, and toughness.

Hardness testing was performed using an Instron B2000 tester (Instron, Norwood, MA, USA) using the HRR scale. Rectangular injection-molded samples of approximately 63.5 mm (length) × 12.7 mm (width) × 3 mm (thick) were tested. A total of four fixed points along the length were taken. Similar to tensile testing, a hardness value was obtained by averaging six tested samples for each POM composite type.

## 3. Results and Discussion

### 3.1. Morphology Analysis Using SEM

#### 3.1.1. Surface Microscopy of Etched PTFE

**Figure 1:**
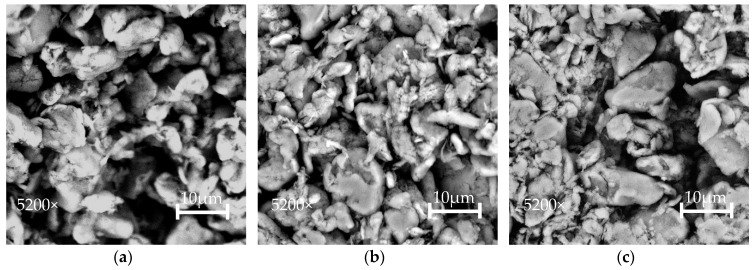
SEM micrographs of PTFE particles etched for (**a**) 2.9 min; (**b**) 10 min; or (**c**) 17.1 min.

The effects of chemical etching on the PTFE surface were studied through SEM using 5200× magnification. The compatibility of POM and PTFE can be increased with this method. Interfacial adhesion of polymeric material to GF provides insights into the mechanical properties of POM/GF/PTFE composites with different PTFE content and PTFE etch time. The surface morphology of PTFE particles etched for 2.9 min, 10 min, and 17.1 min is shown in Figure 1. The surface of a 2.9-min-etched PTFE was generally smooth and particles appeared spherical in shape. With a 10-min etch time, the surface looks rougher, indicating the effects of etching. When PTFE was etched for 17.1 min, the etch depth increased, with porous cavities on the surface. In addition, PTFE microparticles show signs of disintegration. These cavities remain unfilled if the melt polymer is unable to penetrate into the surface imperfections. Investigations have shown that the defluorination depth on the PTFE surface is correlated to the sodium naphthalene etch time. A longer etch time yields a highly porous defluorinated layer. The adhesion mechanism to this porous surface is adhesive mechanical interlocking, which may cause a bond failure by stripping the etched layer away [20]. Hunke et al. [21,22] reported that functional groups in the defluorinated layer are not completely removed even at temperatures of more than 300 °C. This enables the use of treated PTFE particles as potential tribological fillers in high-temperature engineering polymers.

#### 3.1.2. Morphology of POM/GF/PTFE Fractured Surfaces

**Figure 2:**
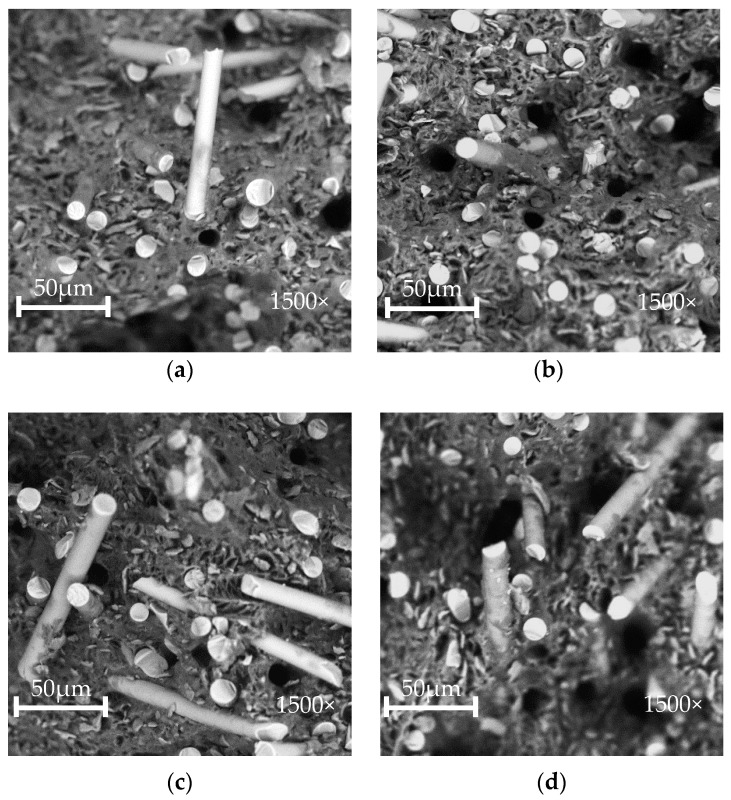
SEM micrographs of fractured surfaces for POM composites with (**a**) 4.0% PTFE etched for 10 min; (**b**) 17.3% PTFE etched for 10 min; (**c**) 9.5% PTFE etched for 2.9 min; (**d**) 9.5% PTFE etched for 17.1 min.

The fractured surfaces of POM/GF/PTFE composites after tensile testing are characterized through SEM using 1500× magnification. The surface morphology of composites blended with different PTFE content and PTFE etch time reveals information on PTFE’s interaction with POM and GF. SEM micrographs show the dispersion of PTFE within POM and the adhesion of POM/PTFE to the surface of GF. Figure 2a,b show the surface morphology of composites with different PTFE contents etched for 10 min. PTFE particles are homogenously dispersed within the POM matrix with 4.0% PTFE content. The POM matrix appears smooth with slight adhesion of polymeric material to GF. Higher PTFE content of 17.3% caused the excessive particles to have non-homogenous dispersion within the POM matrix. Adhesion of POM/PTFE to the GF surface appears significantly higher.

Figure 2c,d compare the effects of 2.9-min and 17.1-min etch times with 9.5% PTFE content. The adhesion of polymeric material to the surface of GF is comparable. PTFE etched for 17.1 min show a slightly higher concentration of PTFE particles within the POM matrix, possibly as a result of disintegration caused by excessive etching (Figure 1c). The change of interfacial bonding force between the matrix and fiber weakens the composite, causing a reduction in both strength and stiffness [28,29,30]. The presence of micro-fillers within the matrix is also known to negatively impact the mechanical properties [8]. Analysis of tensile properties in the subsequent sections quantitatively validates the morphology studies [30,31].

### 3.2. RSM Analysis of Mechanical Properties

The levels of factors and the effect of their interactions on mechanical properties were determined by CCD of RSM. The design matrix of experimental results by tests was planned according to the full factorial designs. Thirteen experiments were performed at different combinations of the factors and the central point was repeated five times. The observed responses along with the design matrix are presented in Table 2. Without performing any transformation on the responses, the results were analyzed by ANOVA. A regression equation provided the relationship of the mechanical properties of the POM composites as a function of PTFE content and PTFE etch time. Tests for the significance of the regression model, the significance of individual model coefficients, and the lack-of-fit are required. An ANOVA table is commonly used to summarize the tests performed.

**Figure 3:**
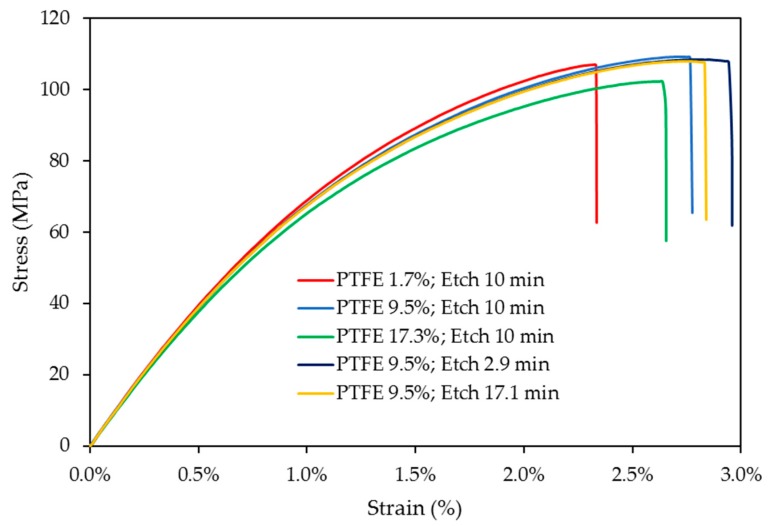
Stress vs. strain curves comparing effects of PTFE content and etch time on POM composites.

The effects of PTFE content and PTFE etch time on the tensile properties of POM composites are displayed in Figure 3. With 10 min PTFE etch time, toughness, represented by the area under the stress vs. strain curves, steadily increased as PTFE content increased from 1.7% to 17.3%. POM composites blended with PTFE etched for 2.9 min and 17.1 min show slightly better toughness than 10-min-etched PTFE. Increase of toughness with higher PTFE content is at the cost of tensile strength and elasticity modulus. When comparing POM/GF/PTFE composites with 9.5% PTFE content at different PTFE etch times, no significant difference in strength and stiffness was observed.

### 3.3. ANOVA Analysis and Model Fitting for Tensile Strength of POM Composite

Table 3 shows the ANOVA table for response surface model for tensile strength. The *F*-value of 24.80 implies the model is significant. There is only a 0.14% chance that an *F*-value this large could occur due to noise. Values of “Prob > *F*” less than 0.05 indicate that the model terms are significant. Values greater than 0.10 indicate the model terms are not significant. The lack-of-fit can also be said to be insignificant. This is necessary as we want a model that fits. The terms A, A^2^, A^2^B, and AB^2^ are significant for the tensile strength of POM composites. The coefficient of determination, *R*^2^, is one of the measures resulting in a reduction of response variability. The *R*^2^ of 0.9720 is very close to 1, in agreement that the model comprises the best fit data. The *R*^2^_adj_ value of 0.9328 suggests the model is sufficient without needing to consider additional terms. An adequate precision measures the signal to noise ratio, and a value greater than 4 is desirable. A precision value of 17.698 indicates an adequate signal. This model can be used to navigate the design space.

The model above was used to predict the tensile strength of POM composites (Y1) as a function of PTFE content (A) and PTFE etch time (B). It can be presented in terms of coded factors, as in the following equation:

(5) $$Y1=107.86-1.81A-0.19B-0.012AB-1.58A^{2}+0.26B^{2}-1.33A^{2}B-1.63AB^{2}$$

**Figure 4:**
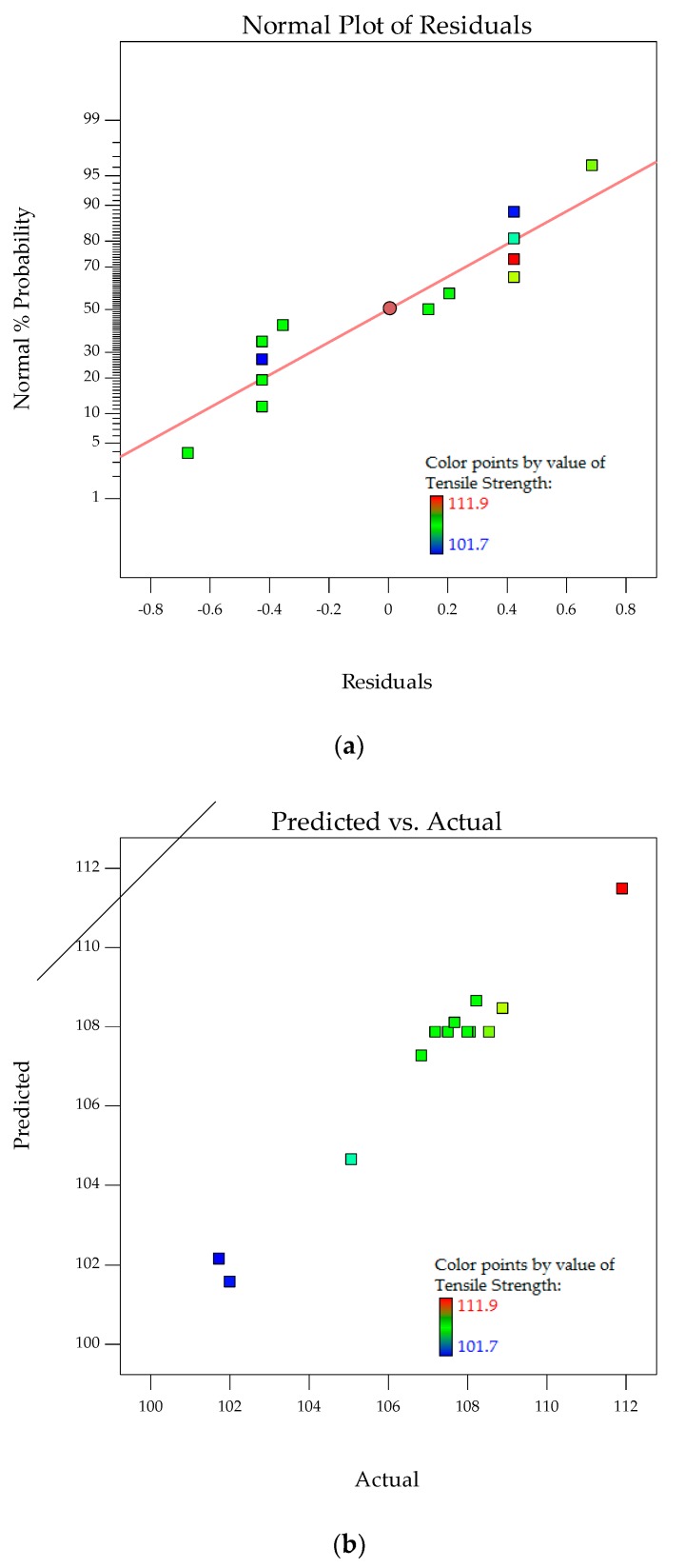
Stochastic error and deterministic portion for tensile strength of POM composites as (**a**) normal probability plot for residuals; (**b**) predicted versus actual values.

Normal probability plot for residuals, i.e., deviation of actual values against the predicted values, analyzes the adequacy of the model by evaluating the data applied in the model. Random and normally distributed residuals indicate none of the predictive information is in the error. The residuals in prediction of response are minimal since they are very close to the diagonal line. Hence, the deterministic portion of the model is good at explaining the response that only the inherent randomness is left over within the error portion [32]. The normal probability plots for residuals and relationship of actual versus predicted tensile strength are shown in Figure 4a,b. The values of *R*^2^ of 0.9720 and *R*^2^_adj_ of 0.9328 along with the residual analysis adequately fit the model to ethe xperimental data.

**Figure 5:**
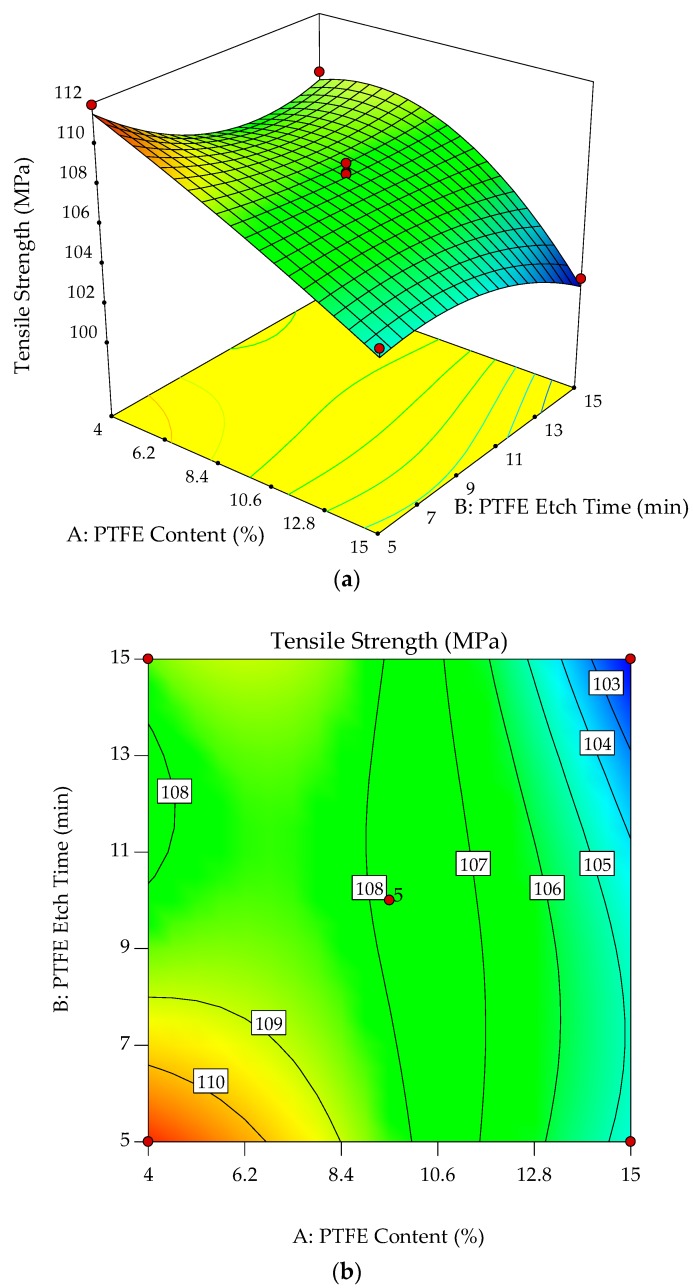
3D response surface plot (**a**) with 2D contour plot; (**b**) of the effects of PTFE content and PTFE etch time on tensile strength of POM composites.

The interaction effects of PTFE content and PTFE etch time on the tensile strength were studied by plotting surface curves. The primary and secondary horizontal axes are the input variables, whereas the vertical axis is the calculated response, i.e., the tensile strength. The 3D surface curves and 2D contour plots from the interactions of these variables are obtained. Figure 5a,b show the dependence of tensile strength on the PTFE content and PTFE etch time.

Generally, the POM composites exhibit continuous decline in tensile strength with increasing PTFE content. As expected, lower strength is obtained at higher PTFE content, with a slight dependence on PTFE etch time. These observations indicate the negative effects of particulate filler on the matrix. Since PTFE is amorphous, its softness leads to a reduction in the strength of the matrix. In addition, the surface of PTFE particles is insufficiently etched when exposed to low etch time, as shown in Figure 1a. The PTFE particles have low surface energy, resulting in poor wettability and inability to bond, leading to a weak interface with the matrix and GF. By increasing PTFE content, these agglomerative PTFE form much larger particles, causing localized stress concentrations [10,11]. As shown in Figure 2b, the adhesion of the polymeric material to GF, coupled with the rather low compatibility of PTFE particles with POM, is unable to bear the stress during the tensile process. PTFE in the form of particles are known to cause agglomeration, affecting the tensile strength, and smaller particulates are able to reduce this effect [8]. Thus, the tensile strength of studied composites decreases as the PTFE content increases. Disintegration of PTFE particles is shown in Figure 1c, and the adhesion to GF observed in Figure 2b contributed to lower strength.

With PTFE content of 4.0% to 9.5%, tensile strength achieves a stable region of approximately 108 MPa, independent of PTFE etch time. At this region, the POM/PTFE appears homogenous, with slight adhesion to the surface of GF, as shown in Figure 2a. The surface of PTFE particles is influenced by etch time but its effect on tensile strength of POM/GF/PTFE composites is rather low. PTFE content of less than 9.5% is insufficient to overcome the GF’s reinforcement. Surface treatment of fluoropolymers changes the chemical composition and increases the surface energy, polarity, wettability, and ability to bond [20]. This stable region is important so that other mechanical properties of POM/GF/PTFE composites can be optimized without compromising strength.

### 3.4. ANOVA Analysis and Model Fitting for Elasticity Modulus of POM Composites

Table 4 shows the ANOVA table for response surface model for elasticity modulus. The *F*-value of 49.85 implies the model is significant. There is a less than 0.0001% chance that an *F*-value this large could occur due to noise. Values of “Prob > *F*” less than 0.05 indicate that model terms are significant. Values greater than 0.10 indicate the model terms are not significant. The lack-of-fit is also insignificant. Only the term A is significant for the elasticity modulus of POM composites. The coefficient of determination, *R*^2^ of 0.9542 is very close to 1, in agreement that the model comprises the best fit data. The *R*^2^_adj_ value of 0.9450 suggests the model is sufficient without needing to consider additional terms. Adequate precision measures the signal to noise ratio and a value of greater than 4 is desirable. The adequate precision value of 30.038 indicates an adequate signal. This model can be used to navigate the design space.

A linear model was used to predict the elasticity modulus of POM composites (Y2) as a function of PTFE content (A) and PTFE etch time (B). It can be presented in terms of coded factors as in the following equation:

(6) Y2=8077.55−202.73A−4.54B

**Figure 6:**
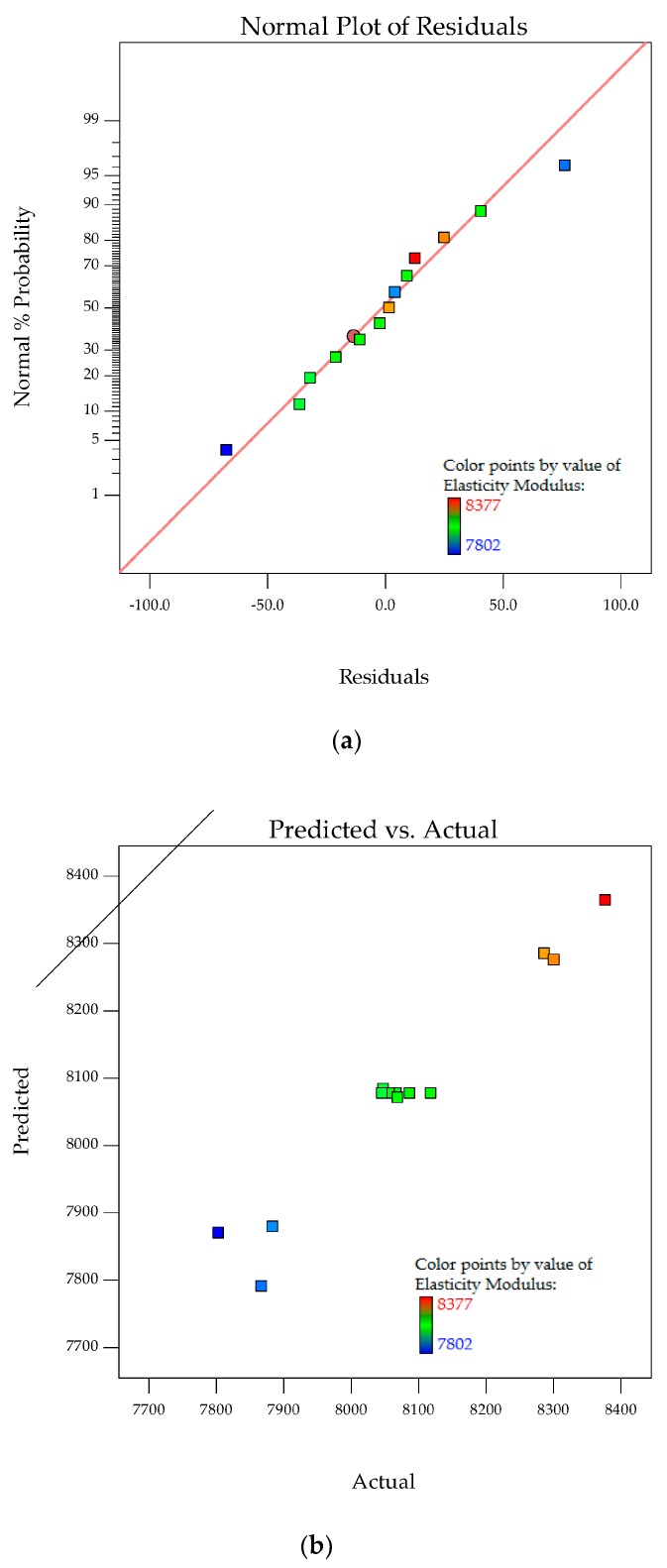
Stochastic error and deterministic portion for elasticity modulus of POM composites as (**a**) normal probability plot for residuals; (**b**) predicted versus actual values.

The normal probability plots for residuals and the relationship of actual versus predicted elasticity modulus are shown in Figure 6a,b. A normal probability plot for residuals analyzes the adequacy of the model by evaluating the data applied in the model for elasticity modulus. Random and normally distributed residuals indicate none of the predictive information is in the error. The residuals in prediction of response are minimal since they are very close to the diagonal line. Hence, the deterministic portion of the model is good at explaining the elasticity modulus with only the inherent randomness left over within the error portion [32]. The values of *R*^2^ of 0.9542 and *R*^2^_adj_ of 0.9450 along with the residual analysis adequately fit the model to the experimental data.

**Figure 7:**
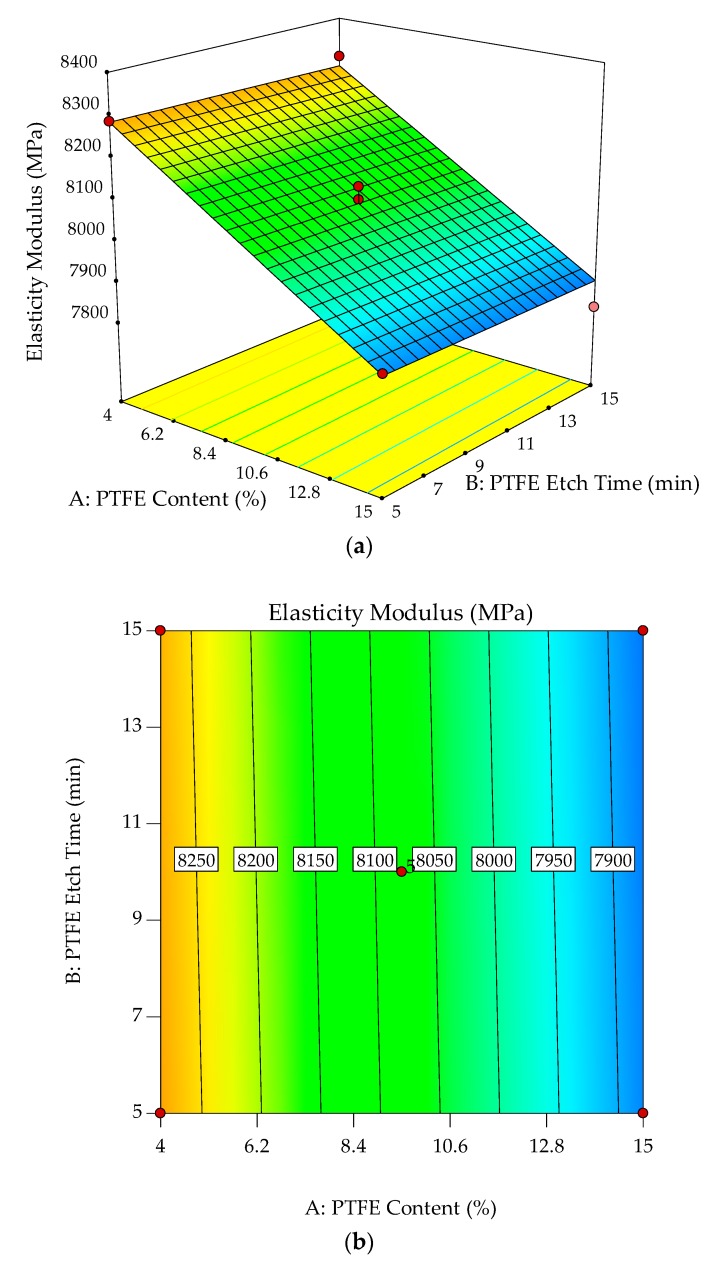
3D response surface plot (**a**) with 2D contour plot; (**b**) of the effects of PTFE content and PTFE etch time on elasticity modulus of POM composites.

The interaction effects of PTFE content and PTFE etch time on the elasticity modulus are studied by plotting surface curves. The primary and secondary horizontal axes are the input variables, whereas the vertical axis is the calculated response, i.e., the elasticity modulus. The 3D surface curves and 2D contour plots from the interactions of these variables are obtained. Figure 7a,b show the dependency of elasticity modulus on the PTFE content and etch time.

The elasticity modulus consistently decreases as the PTFE content increases, independent of PTFE etch time. It is known that micro-fillers in the form of particulate cause a reduction in resistance to deformation. Nano- or micro-sized particles have a strong tendency to agglomerate because of their high surface activity [33,34,35]. Agglomeration takes place during melt blending to form much larger particles, leading to stress concentration sites in composites [8]. In Figure 2b, increasing the PTFE content results in adhesion of POM/PTFE to the surface of GF. With changes to this interface, the reinforcement effects of GF within the composites are compromised. The deterioration in stiffness can also be caused by the amorphousness and softness of PTFE. All of these negatively affect the stress transfer to GF during tensile loading and reduce the resistance to deformation of the composites. Surface-treated PTFE through chemical etching is known to enhance compatibility with the POM matrix when compared to non-treated PTFE [11]. The stiffness of POM composites is greatly influenced by GF due to its adhesion to POM, superior elasticity modulus strength, and high concentration within the composites [16].

### 3.5. ANOVA Analysis and Model Fitting for Toughness of POM Composites

Table 5 shows the ANOVA table for response surface model for elasticity modulus. The *F*-value of 12.59 implies the model is significant. There is only a 0.22% chance that an *F*-value this large could occur due to noise. Values of “Prob > *F*” less than 0.05 indicate that model terms are significant. Values greater than 0.10 indicate the model terms are not significant. The lack-of-fit is also insignificant. This model is desirable as the model that fits. The terms B, A^2^, and B^2^ are significant for the toughness of POM composites. The coefficient of determination, *R*^2^ of 0.8988 is close to 1, in agreement that the model comprises of best fit data. The *R*^2^_adj_ value of 0.8482 suggests the model is sufficient without needing to consider additional terms. An adequate precision measures the signal to noise ratio, and a value of greater than 4 is desirable. The adequate precision value of 15.760 indicates an adequate signal. This model can be used to navigate the design space.

The model above is used to predict the toughness of POM composites (Y3) as a function of PTFE content (A) and PTFE etch time (B). It can be presented in terms of coded factors as in the following equation:

(7) $$Y3=1996.21+36.61A-52.62B-132.43A^{2}+67.10B^{2}$$

**Figure 8:**
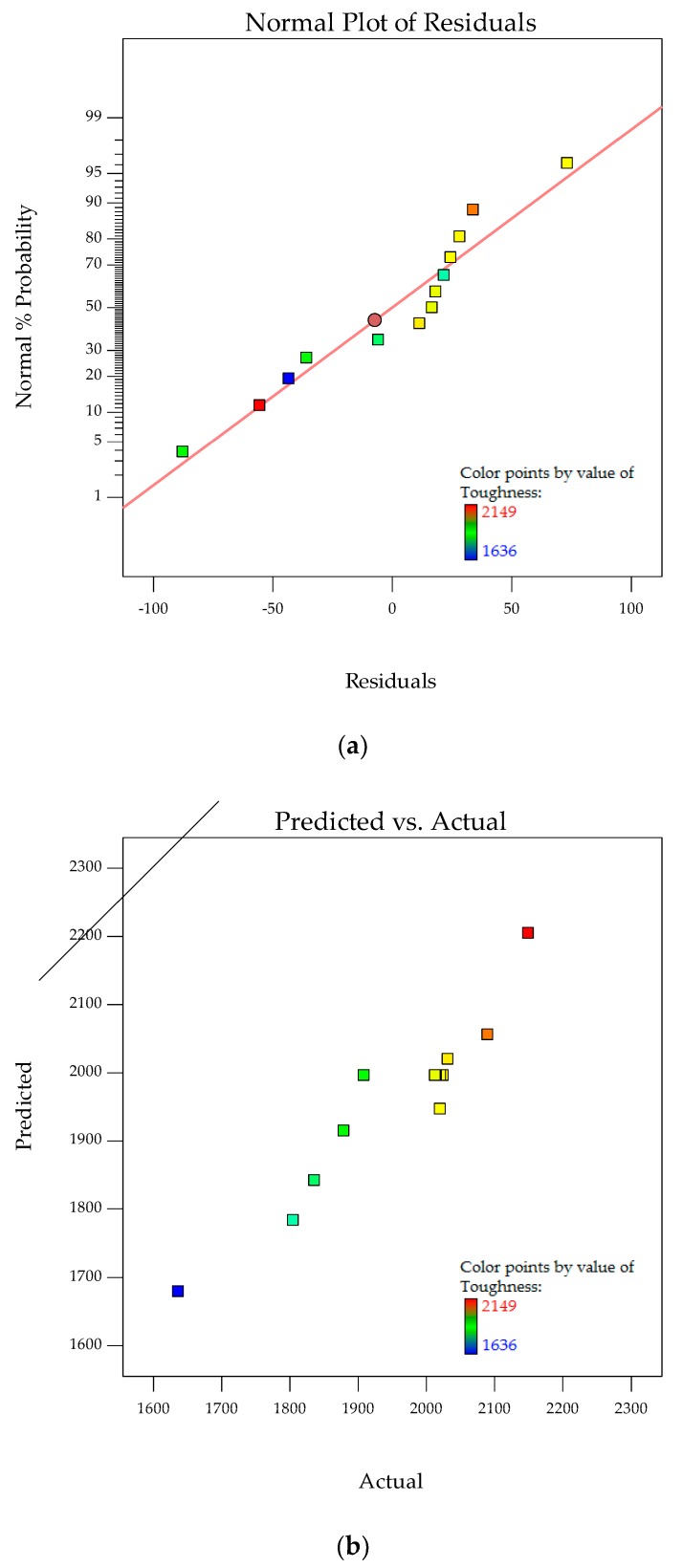
Stochastic error and deterministic portion for toughness of POM composites as (**a**) normal probability plot for residuals; (**b**) predicted versus actual values.

The normal probability plots for residuals and relationship of actual versus predicted toughness are shown in Figure 8a,b. Normal probability plots for residuals analyze the adequacy of the model by evaluating the data applied in the model for toughness. Random and normally distributed residuals indicate that none of the predictive information is in the error. The residuals in prediction of response are minimal since they are very close to the diagonal line. Hence, the deterministic portion of the model is good at explaining the toughness, with only the inherent randomness left over within the error portion [32]. The values of *R*^2^ (0.8988) and *R*^2^_adj_ (0.8482) along with the residual analysis adequately fit the model to experimental data.

**Figure 9:**
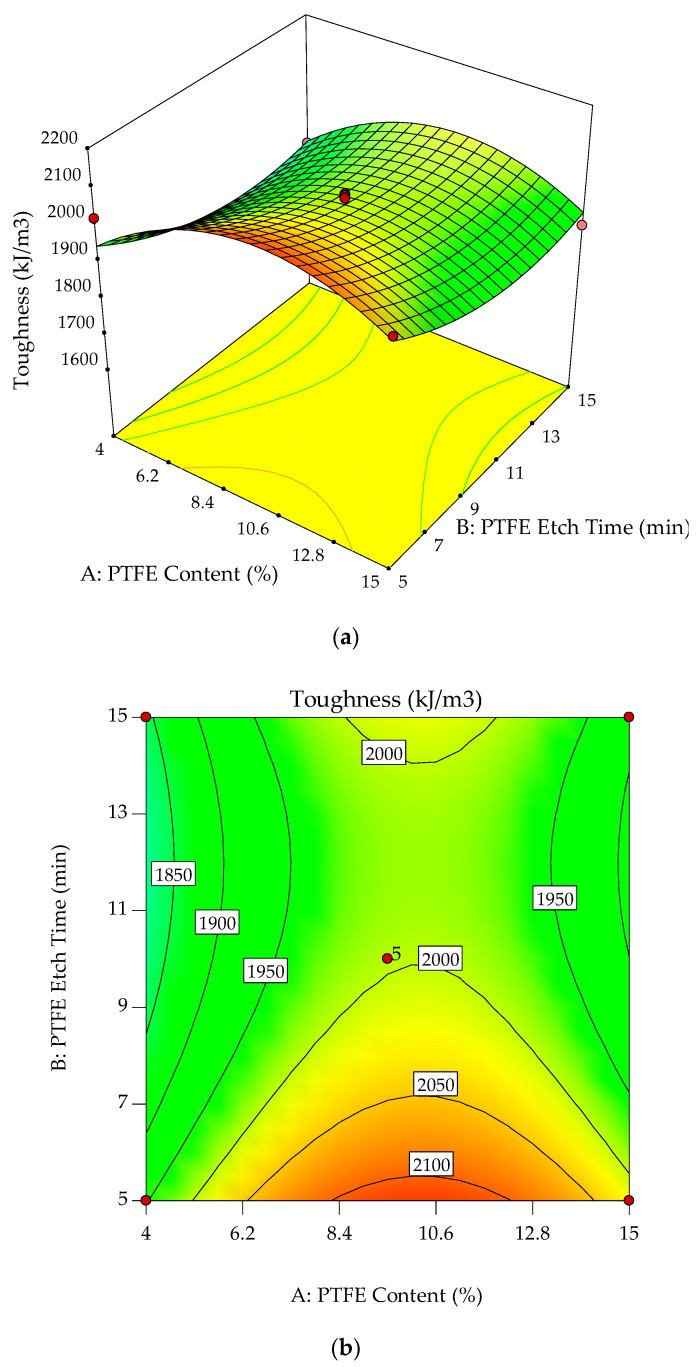
3D response surface plot (**a**) with 2D contour plot; (**b**) of the effects of PTFE content and PTFE etch time on toughness of POM composites.

The interaction effects of PTFE content and PTFE etch time on toughness are studied by plotting surface curves. The primary and secondary horizontal axes are the input variables, whereas the vertical axis is the toughness. The 3D surface curves and 2D contour plots are obtained from the interactions of these variables. Figure 9a,b show the dependency of toughness on the PTFE content and PTFE etch time. The characteristics of toughness and elongation at break are known to be well correlated. As such, only toughness is considered for the study of the mechanical properties of the POM/GF/PTFE composite.

Generally, for any given PTFE etch time, toughness steadily increases with increasing PTFE content and is highest when the PTFE content is approximately 10%. As the PTFE content is continuously increased, the toughness starts to deteriorate. At an optimum PTFE content of 9.5%, the toughness is highest, approximately 2150 kJ/m^3^, with a PTFE etch time of 5 min. With PTFE constant at 9.5%, toughness gradually decreases as the PTFE etch time is increased, reaching a low of 2000 kJ/m^3^ at a PTFE etch time of 10 to 14 min before increasing slightly.

The improvement in toughness indicates there is a synergistic toughening effect of the GF and PTFE on POM. The toughness of polymer composites is affected by the interfacial adhesion between the matrix and fiber. Interaction between POM and GF is improved with the addition of PTFE particles, thus facilitating the mobility of macromolecular chains during tensile testing [18]. However, improvement in toughness is at the expense of a reduction in stiffness and hardness. For tensile strength, the stable region of 4% to 10% PTFE content and 8 to 15 min PTFE etch time, results in the composite’s strength not being compromised by toughness. Hence, composites with PTFE content of 9.5% are important because optimum toughness is achieved. By varying the PTFE etch time, the desired toughness value can be achieved. Similar to the characteristics of strength, this allows other mechanical properties for the POM composite to be optimized without drastically affecting toughness.

### 3.6. ANOVA Analysis and Model Fitting for Hardness of POM Composites

Table 6 shows the ANOVA table for response surface model for hardness. The *F*-value of 68.50 implies the model is significant. There is a less than 0.01% chance that an *F*-value this large could occur due to noise. Values of “Prob > *F*” less than 0.05 indicate that the model terms are significant. Values greater than 0.10 indicate the model terms are not significant. The lack-of-fit is also insignificant. The terms A and A^2^ were significant for the hardness of POM composites. The coefficient of determination, *R*^2^, of 0.9800 is very close to 1, in agreement that the model comprises of best fit data. The *R*^2^_adj_ value of 0.9657 suggests the model is sufficient without needing to consider additional terms. Adequate precision measures the signal to noise ratio and a value of greater than 4 is desirable. The precision value of 26.444 indicates an adequate signal. This model can be used to navigate the design space.

The model above is used to predict the hardness of POM composites (Y4) as a function of PTFE content (A) and PTFE etch time (B). It can be presented in terms of coded factors as in the following equation:

(8) $$Y4=114.35-1.34A-0.02B+0.06AB-0.33A^{2}+0.19B^{2}$$

**Figure 10:**
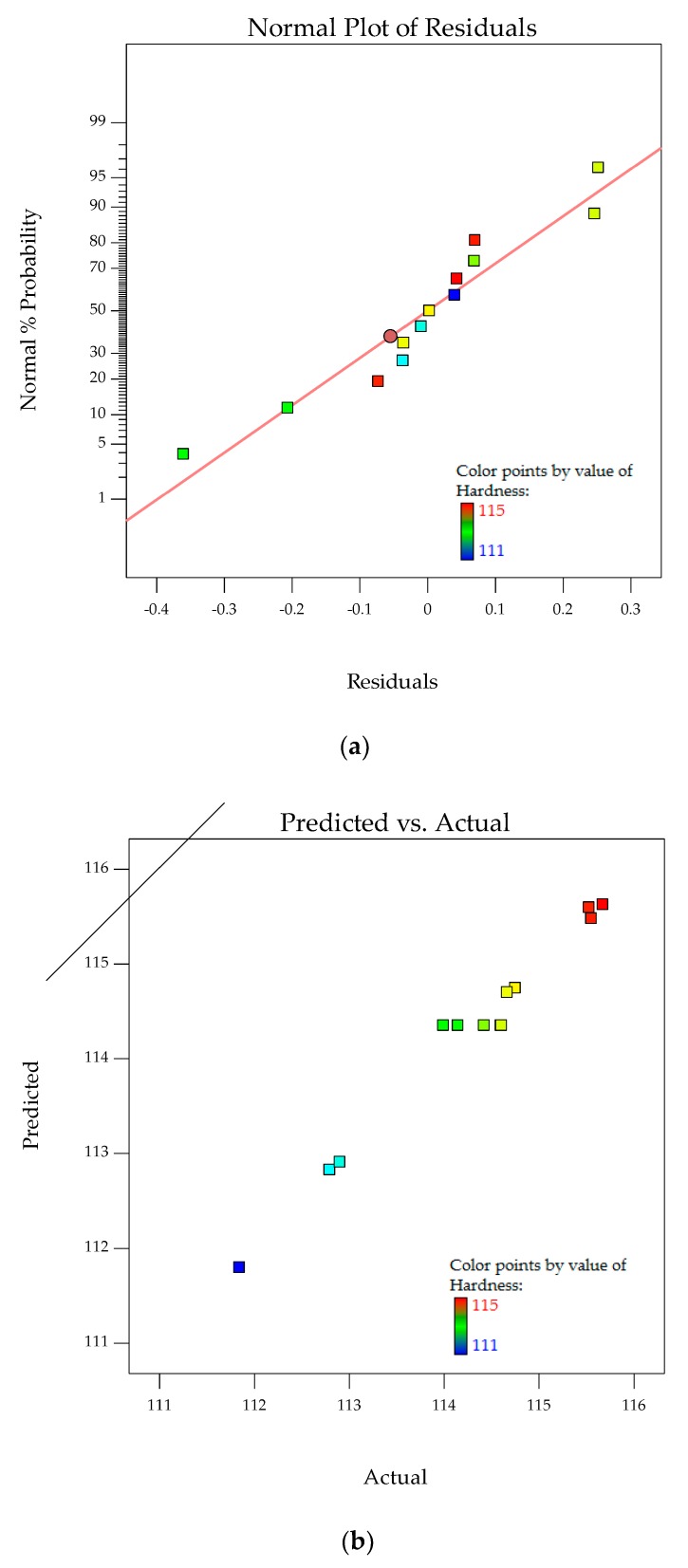
Stochastic error and deterministic portion for hardness of POM composites as (**a**) normal probability plot for residuals; (**b**) predicted versus actual values.

The normal probability plots for residuals and relationship of actual versus predicted hardness are shown in Figure 10a,b. The normal probability plot for residuals analyzes the adequacy of the model by evaluating the data applied in the model for hardness. Random and normally distributed residuals indicate that none of the predictive information is in the error. The residuals in prediction of response are minimal since they are very close to the diagonal line. Hence, the deterministic portion of the model is good at explaining the hardness, with only the inherent randomness left over within the error portion [32]. The values of *R*^2^ (0.9800) and *R*^2^_adj_ (0.9657), along with the residual analysis, adequately fit the model to the experimental data.

**Figure 11:**
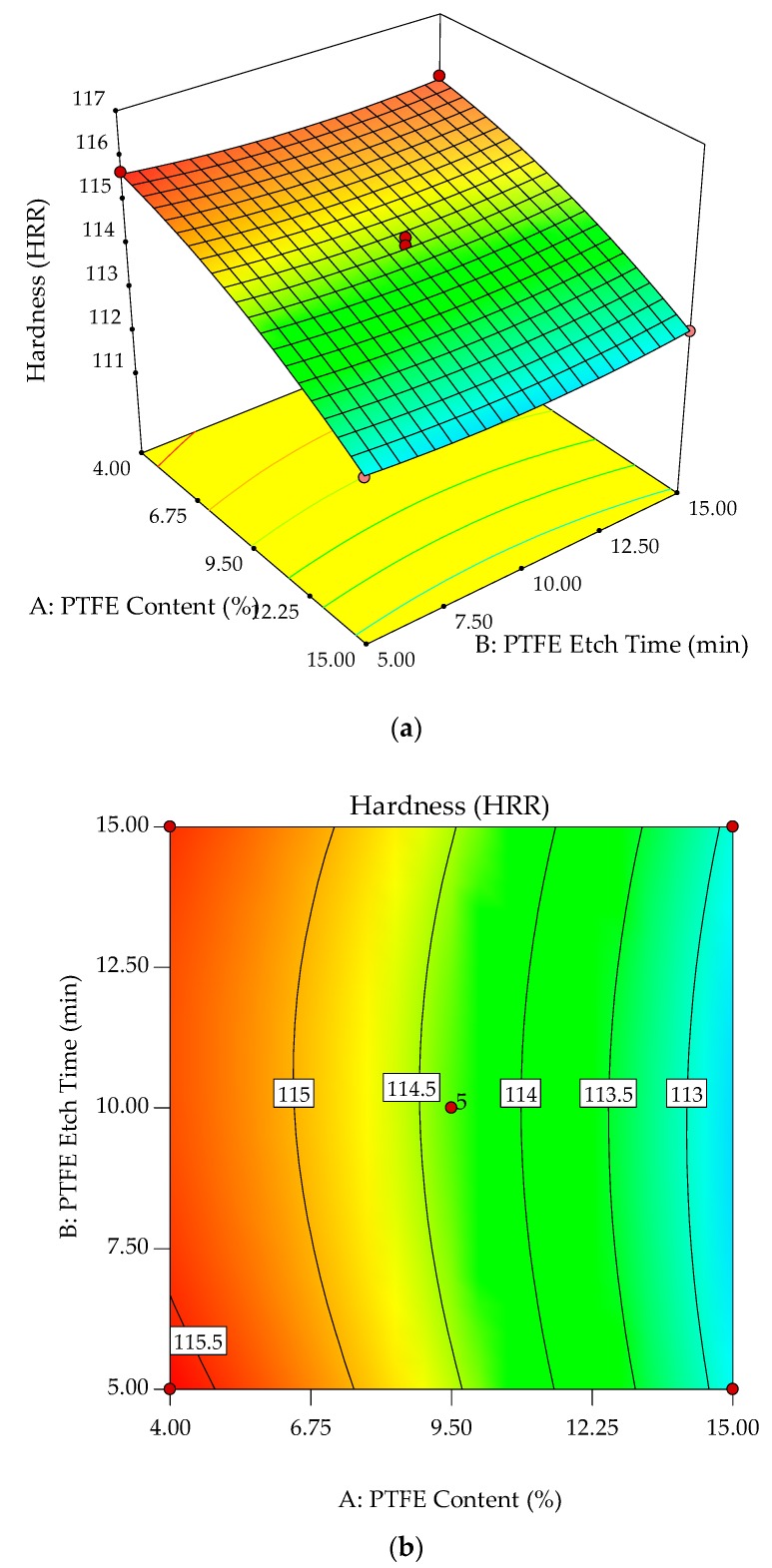
3D response surface plot (**a**) with 2D contour plot; (**b**) of the effects of PTFE content and PTFE etch time on hardness of POM composites.

The interaction effects of PTFE content and PTFE etch time on the hardness are studied by plotting surface curves. The primary and secondary horizontal axes are the input variables, whereas the vertical axis is the calculated response, i.e., the hardness. The 3D surface curves and 2D contour plots are obtained from the interactions of these variables. Figure 11a,b show the dependency of hardness on the PTFE content and PTFE etch time. Hardness decreases as the PTFE content is increased, independent of PTFE etch time. SEM micrographs in Figure 2 show PTFE embedded within the POM matrix and surface of GF adhered with the polymeric material. These observations are similar to the elasticity modulus, where the addition of PTFE filler as microparticles weakens the POM/GF/PTFE composites [8].

### 3.7. Overall Desirability for Mechanical Properties for POM Composites

RSM analysis characterized each mechanical property with varying PTFE content and PTFE etch time. For the optimization study of the mechanical properties, the objective is to simultaneously maximize the POM composite’s strength, stiffness, toughness, and hardness. A useful approach for simultaneous optimization of multiple responses is to use a desirability function. To optimize an using overall desirability function, it is important to formulate the specification for each of the factors and responses shown in Table 7. Specification for tensile strength and toughness are taken as above median of their respective range and of lower importance. For elasticity modulus, the specification is targeted at 8300 MPa with an importance index of 5. As for hardness, it is targeted at 115 HRR, with an importance index of 5. These specifications were selected by referencing the upper limit of the experimental results.

**Figure 12:**
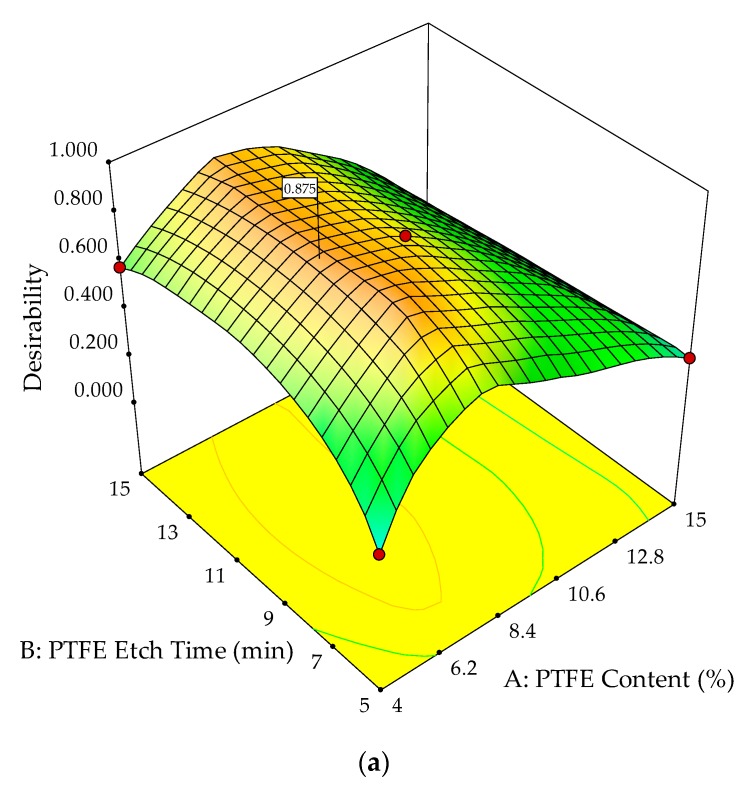

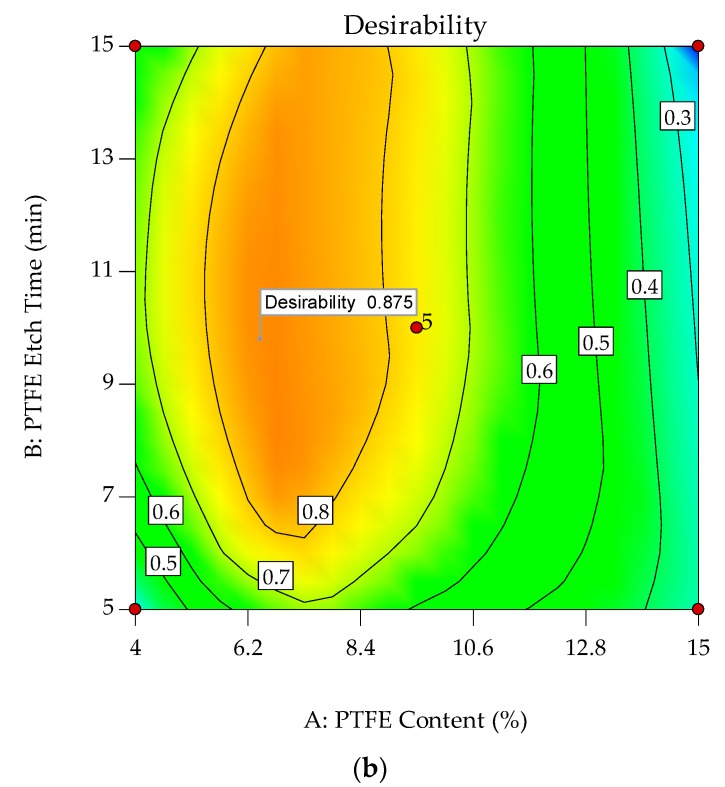
3D response surface plot (**a**) with 2D contour plot; (**b**) of desirability function applied to multiple responses.

Figure 12a,b show the overall desirability function applied to multiple responses simultaneously, i.e., tensile strength, elasticity modulus, toughness, and hardness. The optimum overall desirability (D) of 87.5% was achieved with a PTFE content of 6.5% and a PTFE etch time of 10 min. This optimal point of the system attained by geometric mean maximization was calculated from the individual desirability (d) for each response, as shown in Table 7.

The obtained values for overall desirability (D) and individual desirability (d) are found to be close to the optimum condition of 1. This shows that the POM composites are well optimized. Thus, the mechanical properties of POM composites for this optimized condition are in agreement with the required specifications. The tensile strength is 108.4 MPa, the elasticity modulus is 8190.5 MPa, the toughness is 1937.23 kJ/m^3^, and the hardness is 115.0 HRR.

## 4. Conclusions

The mechanical properties of POM/GF/PTFE composites were optimized by varying the PTFE content and PTFE etch time. As PTFE is known to weaken a polymer composite’s strength and stiffness due to its low surface energy, chemical etching was necessary to improve its compatibility with the POM/GF matrix prior to blending. Therefore, PTFE content and PTFE etch time were important factors in determining the mechanical properties of POM/GF/PTFE composites. The need to control these factors accurately was necessary. SEM analysis correlated the etch time to the defluorination layer on the surface of PTFE particles. Morphology study of fractured surfaces during tensile testing revealed the effects of PTFE content and PTFE etch time on the matrix and GF. The polymeric material adhesion to GF affected the interfacial bond. As a result, strength, stiffness, and hardness were compromised but the toughness improved. The altered GF surface can be an enabler for applications requiring polymer composites with superior strength and tribological properties. Response surface methodology in conjunction with central composite design was used to model the effects of these factors on the strength, stiffness, toughness, and hardness of POM/GF/PTFE composites. Using experimental data and ANOVA, a mathematical model was derived for each response. The normal probability test, significance test, and correlation coefficients determined the significance of fit between the model and experimental data. To optimize the mechanical properties simultaneously, each property was specified and a desirability function was derived. The overall desirability or D global index for the mechanical properties of the POM/GF/PTFE composite was 87.5% when the PTFE content and PTFE etch time were 6.5% and 10 min, respectively. The individual desirability index, d, for tensile strength was 89.6%, the elasticity modulus was 78.0%, the toughness was 82.7%, and the hardness was 100%. Finally, the contour plot for overall desirability showed a wide region of 80% when the PTFE content ranged from 5.0% to 8.0% and the PTFE etch time ranged from 8 min to 13 min.

## Figures and Tables

**Table 1:** List of input variables with their corresponding levels and responses.

Input Variables	Symbol	Axial (−√2)	Low (−1)	Mid (0)	High (+1)	Axial (+√2)
PTFE content (%)	A	1.7	4	9.5	15	17.3
PTFE etch time (min)	B	2.9	5	10	15	17.1

**Table 2:** Central composite design (CCD) in uncoded factors with tensile strength, elasticity modulus, toughness, and hardness as responses.

PTFE Content (A)	PTFE Etch Time (B)	Tensile Strength (MPa)	Elasticity Modulus (MPa)	Toughness (kJ/m^3^)	Hardness (HRR)
1.7	10.0	106.8 ± 1.6	8377 ± 132	1636 ± 102	115.5 ± 0.6
4.0	5.0	111.9 ± 1.6	8287 ± 73	2020 ± 114	115.7 ± 0.3
4.0	15.0	108.9 ± 2.4	8301 ± 110	1836 ± 147	115.6 ± 0.3
9.5	10.0	107.5 ± 1.4	8046 ± 108	2013 ± 87	114.4 ± 0.4
9.5	10.0	108.6 ± 1.7	8087 ± 90	2021 ± 106	114.6 ± 0.7
9.5	17.1	107.7 ± 1.9	8069 ± 129	2090 ± 92	114.7 ± 0.6
9.5	10.0	107.2 ± 1.8	8057 ± 86	2014 ± 97	114.6 ± 0.3
9.5	10.0	108.1 ± 0.9	8067 ± 48	2025 ± 105	114.1 ± 0.3
9.5	2.9	108.2 ± 1.2	8048 ± 48	2149 ± 101	114.8 ± 0.3
9.5	10.0	108.0 ± 1.2	8118 ± 99	1909 ± 119	114.0 ± 0.6
15.0	5.0	105.1 ± 1.9	7884 ± 76	2032 ± 113	112.8 ± 0.3
15.0	15.0	102.0 ± 2.1	7803 ± 75	1879 ± 134	112.9 ± 0.3
17.3	10.0	101.7 ± 3.3	7867 ± 113	1805 ± 150	111.8 ± 0.2

**Table 3:** Analyses of variance (ANOVA) for response surface model for tensile strength of POM composites using CCD.

Source	Sum of Squares	df	Mean Square	*F*	Prob. > *F*	
Model	88.44	7	12.63	24.80	0.0014	significant
A	13.06	1	13.06	25.62	0.0039	
B	0.15	1	0.15	0.30	0.6093	
AB	6.25 × 10^−4^	1	6.25 × 10^−4^	1.23 × 10^−3^	0.9734	
A^2^	17.31	1	17.31	33.98	0.0021	
B^2^	0.46	1	0.46	0.90	0.3853	
A^2^B	3.53	1	3.53	6.92	0.0465	
AB^2^	5.29	1	5.29	10.38	0.0234	
Residual	2.55	5	0.51			
Lack of Fit	1.44	1	1.44	5.17	0.0853	not significant
Pure Error	1.11	4	0.28			
Cor Total	90.99	12				

*R*^2^, 0.9720; *R*^2^_adj_, 0.9328; Adequate precision, 17.968.

**Table 4:** Analyses of variance (ANOVA) for response surface model for elasticity modulus of POM composites using CCD.

Source	Sum of Squares	df	Mean Square	*F*	Prob. > *F*	
Model	3.353 × 10^5^	5	67,068.87	49.85	<0.0001	significant
A	3.288 × 10^5^	1	3.288 × 10^5^	244.38	<0.0001	
B	164.62	1	164.62	0.12	0.7368	
AB	2244.00	1	2244.00	1.67	0.2376	
A^2^	2299.33	1	2299.33	1.71	0.2324	
B^2^	1306.86	1	1306.86	0.97	0.3572	
Residual	9418.45	7	1345.49			
Lack of Fit	6145.04	3	2048.35	2.50	0.1982	not significant
Pure Error	3273.41	4	818.35			
Cor Total	3.448 × 10^5^	12				

*R*^2^, 0.9542; *R*^2^_adj_, 0.9450; Adequate precision, 30.038.

**Table 5:** Analyses of variance (ANOVA) for response surface model for toughness of POM composites using CCD.

Source	Sum of Squares	df	Mean Square	*F*	Prob. > *F*	
Model	2.055 × 10^5^	5	41,102.41	12.59	0.0022	significant
A	10,720.96	1	10,720.96	3.28	0.1128	
B	22,150.59	1	22,150.59	6.79	0.0352	
AB	252.79	1	252.79	0.077	0.7888	
A^2^	1.220 × 10^5^	1	1.220 × 10^5^	37.37	0.0005	
B^2^	31,322.35	1	31,322.35	9.59	0.0174	
Residual	22,851.58	7	3264.51			
Lack of Fit	13,145.38	3	4381.79	1.81	0.2857	not significant
Pure Error	9706.20	4	2426.55			
Cor Total	2.284 × 10^5^	12				

*R*^2^, 0.8988; *R*^2^_adj_, 0.8482; Adequate precision, 15.760.

**Table 6:** Analyses of variance (ANOVA) for response surface model for hardness of POM composites using CCD.

Source	Sum of Squares	df	Mean Square	*F*	Prob. > *F*	
Model	15.55	5	3.11	68.5	<0.0001	significant
A	14.42	1	14.42	317.66	<0.0001	
B	2.20 × 10^−3^	1	2.20 × 10^−3^	0.048	0.832	
AB	0.013	1	0.013	0.29	0.6074	
A^2^	0.74	1	0.74	16.34	0.0049	
B^2^	0.24	1	0.24	5.3	0.0549	
Residual	0.32	7	0.045			
Lack of Fit	0.016	3	5.42 × 10^−3^	0.072	0.9719	not significant
Pure Error	0.3	4	0.075			
Cor Total	15.86	12				

*R*^2^, 0.9800; *R*^2^_adj_, 0.9657; Adequate precision, 26.444.

**Table 7:** Specification for factors and responses with weightage and importance.

Name	Goal	Lower Limit	Upper Limit	Lower Weight	Upper Weight	Importance	Desirability (d)
A: PTFE Content	In range	4	15	1	1	3	1
B: PTFE Etch Time	In range	5	15	1	1	3	1
Tensile Strength	Target = 108.0	101.7	111.9	1	1	3	0.8956
Elasticity Modulus	Target = 8300.0	7802.9	8377.0	1	1	5	0.7797
Toughness	Target = 2000.0	1636.3	2149.4	1	1	3	0.8274
Hardness	Target = 115.0	111.8	115.7	1	1	5	1
